# Dehydrated Human Amnion Chorion Membrane as Treatment for Pediatric Burns

**DOI:** 10.1089/wound.2019.0983

**Published:** 2020-10-21

**Authors:** Natasha Ahuja, Richard Jin, Colin Powers, Alexandria Billi, Kathryn Bass

**Affiliations:** ^1^ Department of Surgery, University at Buffalo Jacobs School of Medicine and Biomedical Sciences, Buffalo, New York, USA.; ^2^ Department of Pediatric Surgery, John R. Oishei Children's Hospital, Buffalo, New York, USA.

**Keywords:** dehydrated human amnion chorion membrane allografts, pediatric burns, pediatric wound care

## Abstract

**Objective:** Pediatric burns are a major source of injury and in the absence of adequate care can lead to lifelong functional loss and disfigurement. While split thickness skin autografts are the current standard of care for deep partial and full-thickness burns, this approach is associated with considerable morbidity. For this reason, alternative skin substitutes such as allografts have gained interest.

**Approach:** In the present study, we present a case series of 30 children with various types of burns treated with dehydrated human amnion chorion membrane (dHACM).

**Results:** We show that treatment with dHACM is associated with an excellent rate of healing comparable to split thickness skin grafts with less rate of hypertrophic scar and contracture.

**Innovation:** Treatment with dHACM is particularly attractive as it consists of many tissue regenerative factors, such as growth factors and immune modulators, thus it will reduce the risk of scaring.

**Conclusion:** While dHACM is associated with an increased upfront cost, treating patients with small to moderate-sized burns with dHACM in their regional centers works to decrease downstream costs such as management of prolonged pain from donor-site morbidity, revisional surgeries from scar and contractures of split thickness grafts, and avoiding the cost of transfer to higher level centers of care. Our findings challenge the current standard of care, suggesting that dHACM provides an alternative to the current use of split thickness skin grafting and is a safe, feasible, and potentially superior substitute for the management of small to moderate total body surface area partial and full-thickness pediatric burns.

**Figure:**
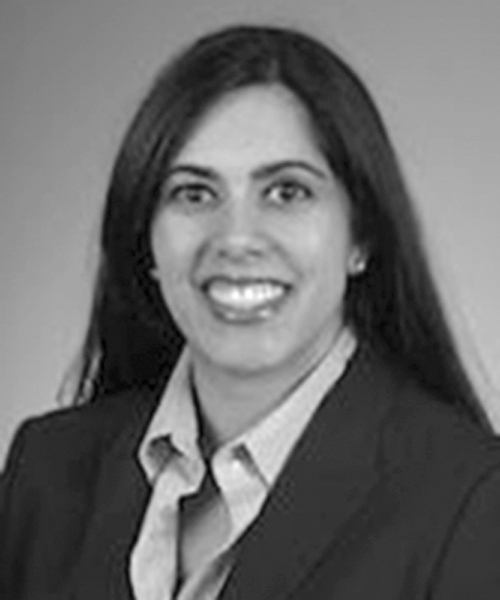
**Natasha Ahuja, MD**

**Figure:**
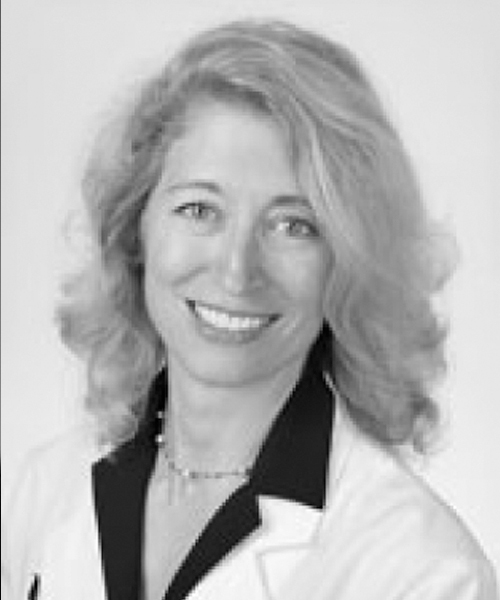
**Kathryn Bass**

## Introduction

Burn injuries are a prevalent and devastating cause of unintentional morbidity and mortality in the United States. In 2015, burn injuries resulted in 356,000 visits to the emergency departments.^1^ The pediatric population (age 1–15) is particularly vulnerable, accounting for 23.5% of total burn cases, second only in prevalence to the adult age group (age 20–59), accounting for 55%.^2,3^ For severe burn wounds, patients are often transferred to dedicated burn centers. Referral criteria to burn centers include extensive burns; that is, partial thickness burns >10% total body surface area (TBSA) as well as burns to face, hands, feet, and genitalia. American Burn Association (ABA) data from 2008 to 2017 demonstrated that 67% of burns treated in ABA centers were <10% TBSA involving the face, hands, feet, genitalia, perineum, or major joints.^3,4^ This population does not require the significant fluid resuscitation and critical care of specialized burn centers and presents a target opportunity for the use of dehydrated human amnion chorion membrane (dHACM) in children. Keeping families together in their own regional hospitals without the burden of travel to receive care provides a major benefit to families and their social support during treatment and recovery.

The management of severe pediatric thermal burns is varied and dependent on the severity of burn. In the absence of adequate treatment, burns can result in significant scarring, contracture, and loss of function.^5^ The current standard of treatment for deep partial thickness and full-thickness burns is surgical debridement of nonviable tissue followed by split thickness skin grafting (STSG).^5,6^ Although widely used in adults, skin autografts pose additional challenges to the pediatric population. An autograft allows coverage of the burn wound, which will reduce pain, fluid loss, and risk of infection at the primary site; but, at the cost of creating a secondary wound that is reported by patients as a greater source of pain than the primary injury, and creates another site for potential fluid loss, infection, and poor cosmesis.^5^ A variety of allograft products and acellular dermal substitutes are available for the treatment of burns and have demonstrated some efficacy in the treatment of pediatric burn wounds.^5,6^ An ideal pediatric burn wound graft should be infection resistant, cost effective, available widely with a long shelf life, lack antigenicity, be flexible to the depth and contour of the wound, durable without need for replacement, and easy to apply and secure. We chose to focus on dHCAM as an alternative to allograft due to its role in modulating immunologic parameters, allowing burn wound repair and meeting many of the objectives listed above.^7^

## Clinical Problem Addressed

Wound healing is a highly dynamic process reliant on well-coordinated phases of repair. While repair is determined by the ability of diverse stem cell populations to repopulate the damaged niche, the efficacy of tissue regeneration is highly driven by the immune process.^8–15^ Decoupling the temporal or qualitative nature of immune responses in the context of wound healing leads to chronic wounds or aberrant regeneration.^12,16,17^ Regardless of the insult, the immunity in most tissues follows a very stereotypic response, leading to efficient repair. However, a number of factors can inhibit this response.

After an injury, damaged tissue and tissue-resident macrophages secrete a number of chemokines that facilitate the rapid influx of innate immune cells, including neutrophils and inflammatory monocytes.^11,18–23^ Natural killer cells and T cells also enter the tissue and act in concert to drive the differentiation of inflammatory monocytes into inflammatory macrophages.^14,19,21,24^ Collectively, these cells constitute the acute inflammatory phase of repair necessary for induction of the immune response, activation of stem cell populations, and clearance of necrotic debris.^14,19–21,23,25^ As the repair proceeds, wound healing enters a regenerative phase marked by migration and proliferation of tissue-resident stem cells and ultimately, tissue remodeling.^20,26–29^ These phases are mirrored by a transition from inflammatory macrophage-mediated repair to restorative macrophage-mediated repair as well as the function of regulatory T cells.^9,14,17,23,30–33^ Collectively, both immune subsets play a dual role in quelling overt inflammation and producing growth factors and tissue remodeling factors such as members of the epidermal growth factor and matrix metalloproteinase families.^9,20,23,34–36^ While the factors that directly mediate the transition toward the regenerative phase are not completely understood, various immune modulatory cytokines have been implicated, such as IL-10.^37^

dHACM has been shown to reduce scar tissue formation and enhance wound healing in diabetic foot ulcers, venous leg ulcers, and select burn cases.^7,38–40^ This therapeutic benefit may be imparted by the ability of human amniotic membrane to not only provide a biologic barrier for the wound but also modulate inflammation and growth factors to foster an enriched regenerative tissue environment. The human amniotic membrane is composed of two conjoined membranes, amnion and chorion. The amnion faces the fetus and consists of organized collagen-rich extracellular matrix, viable cells, regulatory proteins, and signaling molecules.^41^ It is comprised of five distinct layers: the epithelium, basement membrane, compact layer, fibroblast layer, and spongy layer.^41^ The chorion is uterus facing, three to four times thicker than the amnion, and comprised of a reticular layer, basement membrane, and trophoblast layer.^41^ Importantly, amniotic membrane grafts harbor a number of developmental cytokines that play important roles in tissue formation.^42^ Together, these factors aid in development as the tissue must grow and expand without scar tissue formation to successfully carry the growing fetus. Previously, 226 growth factors, cytokines, and chemokines have been identified in dHACM.^43–47^

Given the ability of dHACM to influence multiple modalities of repair, we sought to determine the clinical efficacy of using dHACM in the treatment of pediatric small and moderate TBSA partial and full-thickness burns at risk of functional deficits.

## Materials and Methods

We performed a retrospective chart review between 2017 and 2018, and selected 30 burns that involved the face, hands, feet, genitalia, and major joints occurring in children between 0 and 18 years presenting to our Children's Hospital. Patients with >20% TBSA burns were transferred to a Pediatric Burn Center. The patients were divided into three groups, including superficial partial thickness, deep partial thickness, and full-thickness burn injuries. Data collected included patient age, gender, mechanism of burn, % body surface area, body region, number of weeks to complete healing, and reported pain after dHACM grafting. The literature was reviewed for time to healing of STSG based on depth of burn wound.^48–55^ These results were tabularized, and were compared with rates of healing for dHACM grafts dependent on depth of burn wound.

All burns were treated in the operating room (OR) with a primary sharp debridement of grossly dead and devitalized tissue followed by an ultrasonic debridement of the wound bed until fine bleeding was provoked. Hypochlorous acid was used to wash the surface of the wound before application of the dHCAM graft to each site. The grafts were hydrated by the wound surface and became immediately adherent, then additionally held in place by a porous, emollient noncontact layer sutured around the wound. Surgical lubricant was layered onto the noncontact layer, and then the wound was dressed with nonadherent gauze pad and soft roll. To secure the dressing as childproof for 1 week, an elastic bandage and self-adherent wrap were applied. In the case of hand burns, to immobilize the hand from movement, after applying the soft roll, a fiberglass cast was applied to the mid upper arm over a 90° bent elbow.

Patients were seen weekly for dressing changes. The outer dressing was removed down to the emollient noncontact layer, and hypochlorous acid was used as a wound cleanser to remove any excessive exudate that had extruded through the porous noncontact layer. Depending on the age, some of these cases were taken to the OR to assist the child with sedation to allow for thorough cleaning and redressing of the burn. At week 2, the emollient noncontact layer was removed and only replaced if there was still burn wound surface that had not re-epithelialized. Time to complete burn wound healing and patient satisfaction was assessed at each weekly visit. Pain scores were assessed weekly using the Wong-Baker FACES score scale, and scars were assessed using the Vancouver Scar Scale (VSS) at the 6-week appointment after completion of the re-epithelialization. Any complications encountered were charted weekly.

## Results

All burns treated in our center, with the exception of two cases (which were 15% and 17% TBSA), were treated with maintenance fluid on presentation, did not require significant fluid resuscitation, that is, Parkland formula, and were managed as outpatients. Pain was exceptionally well controlled in all cases without the use of narcotics by using scheduled Acetaminophen and Ibuprofen alternating at 3-hr intervals, while the child was awake over the first 48 h. Parents and caregivers were instructed to use this routine thereafter as needed. Parents reported good pain control with this approach and at weekly intervals, 0–1 points was achieved for all patients on the Wong-Baker FACES scale.

Dressings remained intact for the majority of children. For the few who were able to deconstruct our elaborate multilayer wrap, inspection in the emergency department or in clinic revealed that the grafts were held in place with emollient layer intact. Unlike skin grafting where the percentage take is variable across different wound types and fails to engraft on bone or tendon, dHACM is capable of stimulating granulation tissue and subsequent epithelialization over a larger variety of wound types, including bone and tendon.

While gathering data, we looked at the average time to the OR for debridement and graft placement after initial injury. Not all wounds presented immediately after injury creating a wide variation in time to surgical debridement. In superficial partial thickness burns (*n* = 3), time to OR averaged 4.7 days (±3.5). Deep partial thickness burns (*n* = 22) averaged 2.5 days (±2.6), while full-thickness burns (*n* = 5) took an average of 10 days (±8.7). The results of the full-thickness burns were skewed by three different cases where the patients presented 2–3 weeks after initial injury in the office with wounds that were stalled. Typically, patients who presented immediately after the initial injury to the emergency department with a full-thickness burn were taken to the OR the next day. Tables 1–3 provide the specifics of the patients within this study, demonstrating mechanism, location, and time to heal.

**Table 1.** Superficial partial thickness burns

Age/Gender	Mechanism of Burn	Body Region	%TBSA	No. of Weeks to complete healing	VSS	Complications
14-Month male	Contact heat burn	Left palmar surface of second to fifth digits	1	3.5	1	Hypertrophic scarring
2-Year female	Spilled hot coffee	Chest (including b/l nipples), right arm	6	3	0	
18-Month female	Spilled hot oil	Right buttock, right posterior thigh, posterior LLE, left medial ankle	6	3	1	

LLE, left lower extremity; TBSA, total body surface area; VSS, Vancouver Scar Scale.

**Table 2.** Deep partial thickness burns

Age/Gender	Mechanism of Burn	Body Region	%TBSA	No. of Weeks to Complete Healing	VSS	Complications
17-Month male	Unknown mechanism	Left fingers, circumferential	<1	3	1	Hypertrophic scarring
11-Month male	Dunked hand into hot water	Left palm and digits	1	2.5	0	
17-Year male	Fire burn from gasoline	Right hand	1	2.5	0	
16-Month male	Placed hand on cast iron stove	Left hand	1	4	0	
13-Month male	Touched open oven door	Bilateral hands	2	4	0	Fungal infection noted after third dressing change
17-Month male	Ramen noodle spill	RUE	3–4	4	1	
8-Year male	Placed into hot water tub	Bilateral feet (circumferential around digits)	3	5	1	
3-Year female	Fall into fire embers while camping	Lower back and left arm	3	4	1	
7-Month male	Crawling on hot furnace	Bilateral hands, abdomen	4 (1% each hand, 2% abdomen)	3.5	1	
12-Month female	Curling iron	LLE	4	3	0	
16-Year female	Spilled hot tea	Labia majora, bilateral inner thighs	5	4	0	
10-Month male	Pulled hot tea onto self	RUE	5	2.5	0	
5-Month female	Scald burns from hot water in tub	Bilateral thighs, groins, perineum, lower abdomen	5	3	0	
17-Month male	Fall onto hot coals	Bilateral hands and knees	5	2.5	0	
2-Year male	Spilled tea on self	Bilateral LE	6 (5% on right leg, 1% on left)	2	1	Hypertrophic scarring
12-Year female	Grease fire	Face, right arm, left leg	8	4	2	Scratches due to itching, dog scratched left leg
9-Month male	Unknown mechanism	Face, left neck, auricular area	10	4	0	
4-Week male	Accidental hot water	LLE, including knee joint, foot, and perineum	10	2.5	0	
17-Month male	Spilled hot tea	Chest, bilateral arms	10	4	1	Hypertrophic scarring
18-Month female	Pulled hot tea onto self	LUE, chest (including nipple), abdomen	12	3	2	Admitted POD#5 for fever and vomiting, but no signs of infection; early hypertrophic scarring-resolved compression dressing
15-Month male	Pulled hot water pot	Face, chest, and left arm/axilla	15	1.5	1	During OR, patient developed hypothermia and was recovered in PICU for warming; complained of itching
12-Year female	Crockpot water fell onto patient	Right thigh, RUE, abdomen	17	2	0	

LE, lower extremity; LUE, left upper extremity; OR, operating room; RUE, right upper extremity.

**Table 3.** Full-thickness burns

Age/Gender	Mechanism of Burn	Body Region	%TBSA	No. of Weeks to Complete Healing	VSS	Complications
17-Month male	Fall onto wood burning stove	Bilateral finger pads and palms	1–2	5	1	
3-Year female	Treadmill burn	Bilateral hands with exposed tendon at PIP joint	2	2	5	Scar contracture
10-Year male	Gas can explosion	Left ear and left LE	3	9	0	Friction blisters, poor wound care-fungal infections
13-Month female	Pulled hot tea	RUE	3	4	1	Minor hypertrophic scarring, poor follow-up as patient was out of town for a month
3-Year female	Spilled hot soup	Left upper thigh and suprapubic region	8	5	0	Emergency department visit: concern for infection, none observed

PIP, proximal interphalangeal.

**Figure 1.**
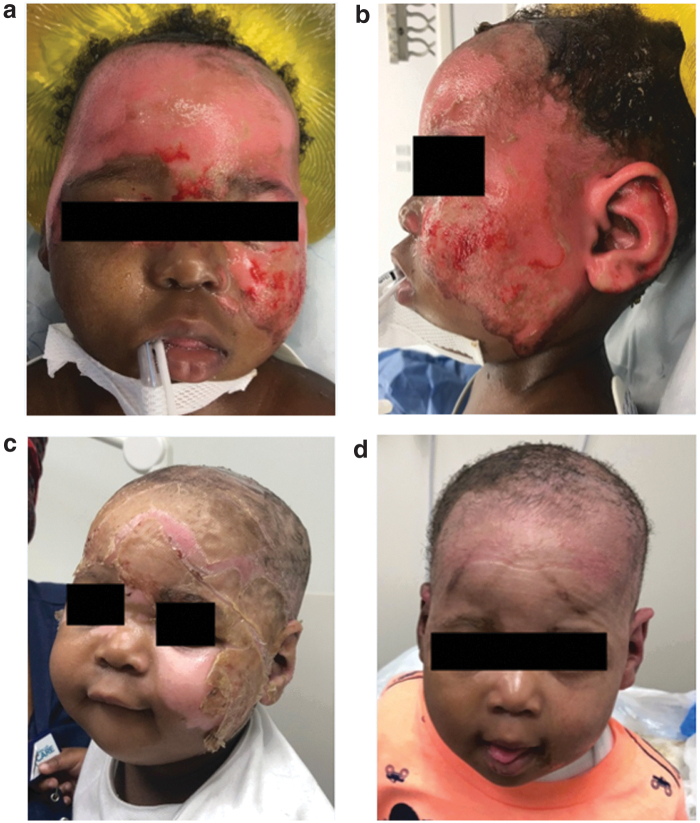
Superficial partial thickness facial burn with rapid return to natural skin tone after dHACM graft. **(a, b)** Initial debridement, **(c)** postop day 7, and **(d)** complete healing. dHACM, dehydrated human amnion chorion membrane.

**Figure 2.**
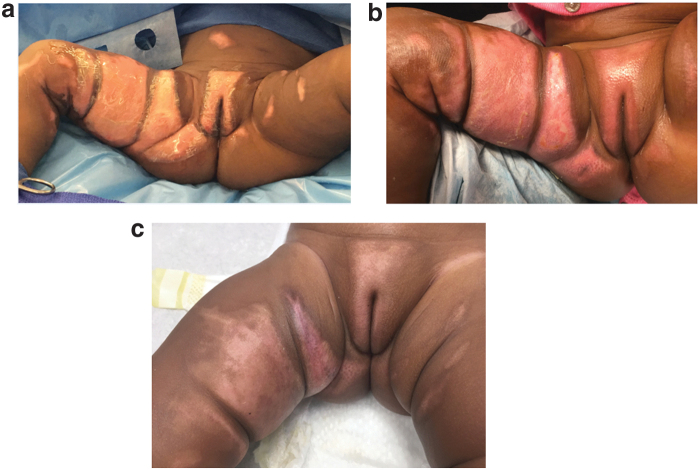
Deep partial thickness genitalia burn. **(a)** Initial debridement after grafting, **(b)** postop day 7, **(c)** complete healing at week 3 with normal skin quality no scarring.

**Figure 3.**
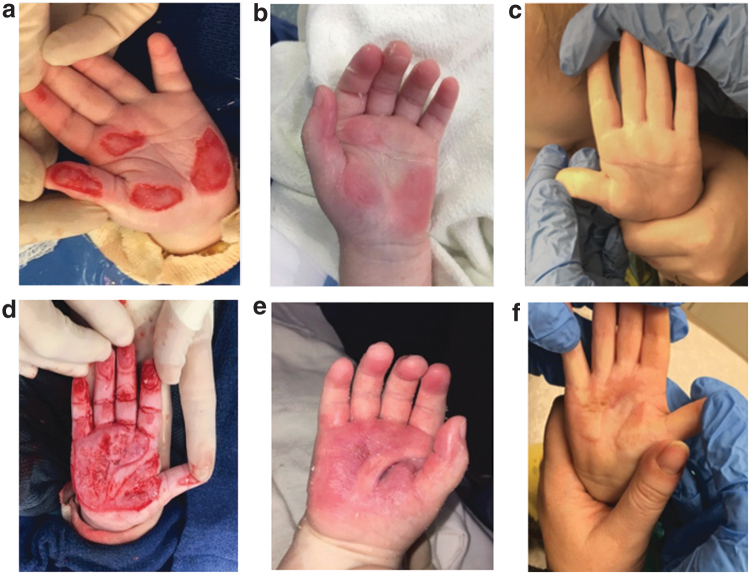
Full-thickness hand burns to fingers bilaterally **(a, d)** initial debridement, **(b, e)** complete healing postop day 38, **(c, f)** postop day 115 with no contractures and minimal scarring.

The average surface area and the average number of days to complete healing were calculated. For superficial partial thickness burns, the average surface area affected was 31.8 cm^2^ (±18.9) with an average time to complete healing of 22 days (±2.6). The average surface area affected for deep partial thickness burns was 167.8 cm^2^ (±220.5) with an average time to complete healing of 23 days (±6.2). Finally, the average surface area affected for full-thickness burns was 75.9 cm^2^ (±58) with an average time to complete healing of 49 days (±14.9). The time to healing in the full-thickness burns was skewed as there was one patient who took 63 days to heal as a result of missed clinic appointments and poor compliance to wound care and immobilization. Table 4 summarizes the results we found in this study. Figures 1–3 demonstrate findings for the various burns at initial injury, week 1 after dHACM grafts, and complete healing for each burn depth group. Consent was obtained from the children's parents for their photographs to be used for this article.

**Table 4.** Summary statistics

Burn Depth Thickness	Sample Size	Average Time to OR After Initial Injury (Days ± SD)	Average Time to Complete Healing (Days ± SD)	Average Surface Area (cm^2^ ± SD)	Average VSS
Superficial partial	3	4.7 ± 3.5	22 ± 2.6	31.8 ± 18.9	0.67 ± 0.58
Deep partial	22	2.5^a^ ± 2.6	23 ± 6.2	167.8 ± 220.5^b^	0.59 ± 0.67
Full	5	10^c^ ± 8.7	49^d^ ± 19.8	75.9 ± 58	1.4 ± 2.07

^a^ One case presented as a chronic wound 4 weeks after the initial burn injury.

^b^ Standard deviation is skewed by one case where the burn was 1,030 cm^2^.

^c^ Three of these cases presented 12, 14, and 21 days after initial injury.

^d^ One case took 63 days to heal secondary to missed clinic appointments and poor compliance with wound care.

VSS was applied to burn wounds at the completion of re-epithelialization and at a 6-week follow-up scar evaluation. Most patients had scores of 0–2 at 6 weeks with normal vascularity and pliability upon re-epithelialization. There were also a few cases with hypopigmentation and a few with hypertrophic scars (HTS) that were <2 mm in height. However, there was one case with a score of 5 as the patient developed minor contractures after 6 weeks.

While STSG is the current standard, it is associated with known complications, including the development of contractures and subsequent need for revisional surgery.^48^ The dHACM results describing time to healing and complications with HTS/contractures were compared with results from previously described STSG studies.^49–56^ The time to healing for dHACM on average was less than STSG, as it took 15–21 days to heal the various depths of burns while STSG took on average >21 days, as demonstrated in Table 5. The rates of HTS and contractures of dHACM grafts were compared with eight representative studies describing STSG. Deep and full-thickness burn injuries face the greatest risk of HTS and contractures. For these injuries, dHACM resulted in 18.5% rate of HTS and contractures, in comparison with STSG studies, which resulted in 57% and 64.1%, see Cubison^55^ and Rotatori,^49^ respectively (Table 6).

**Table 5.** Time to healing of dehydrated human amnion chorion membrane versus split thickness skin grafting

		dHACM				STSG		
						Kishikova et al.^56^	Cubison et al.^55^	Lonie et al.^54^
Time to Healing		Superficial Partial Thickness	Deep Partial Thickness	Full Thickness	Total	Superficial Partial, Deep Partial, Full Thickness	Deep Partial/Full thickness	N/A
1–14 days	*n* (%)	0 (0)	3 (13.6)	1 (20)	4 (13.3)	1 (7.1)	16 (18.6)	0 (0)
15–21 days	*n* (%)	2 (66.7)	9 (40.9)	0 (0)	11 (36.7)	1 (7.1)	16 (18.6)	1 (4)
22–30 days	*n* (%)	1 (33.3)	9 (40.9)	1 (20)	11 (36.7)	0 (0)	22 (25.6)	10 (40)
>30 days	*n* (%)	0 (0)	1 (4.5)	3 (60)	4 (13.3)	12 (85.7)	32 (37.2)	14 (56)
Total No. of patients		3	22	5	30	14	86	25
Median (days)		15–21	15–21	>30	15–21	>30	22–30	>30
Mean (days)		22	15.6	35	19.4	46.1	—	—

dHACM, dehydrated human amnion chorion membrane; STSG, split thickness skin grafting.

**Table 6.** Complications of dehydrated human amnion chorion membrane versus split thickness skin grafting

Study	Burn Depth	Patients (HTS/Contracture)	Site	%
dHACM				
	Superficial partial thickness	3 (1)	Primary	33.3
	Deep partial thickness	22 (3)	Primary	13.6
	Full thickness	5 (2)	Primary	40.0
	Total	30 (6)	Primary	20.0
STSG				
Kishikova *et al.*^56^	Superficial partial, deep partial, full thickness	14 (7)	Primary	50.0
Cubison *et al.*^55^	Deep partial/full thickness	86 (49)	Primary	57.0
Lonie *et al.*^54^	N/A	25 (21)	Primary	84.0
Grossova *et al.*^53^	Superficial partial, deep partial	24 (5)	Primary	20.8
Othman Al Shlash *et al.*^50^	N/A	56 (7)	Primary	12.5
Park *et al.*^51^	N/A	210 (25)	Primary	11.9
Chandrasegaram and Harvey^52^	N/A	126 (34)	Primary	27.0
Rotatori *et al.*^49^	Deep partial/full thickness	237 (152)	Donor	64.1

HTS, hypertrophic scars.

## Discussion

Pediatric burns are a cause of long-term morbidity and can result in a lifetime of functional and esthetic disfigurement. The current standard of care for small to moderate TBSA deep partial and full-thickness burns calls for surgical debridement and rapid autografting, often at a dedicated burn center.^5,6^ However, this approach is subject to a number of challenges that adversely affect both the efficacy and cost of care. We present a case series of 30 children who demonstrate the utility of dHACM as an efficacious substitute for autografts in the management of complex pediatric burn wounds. The use of allografts as skin substitutes such as dHACM circumvents the need to generate a donor site, thereby eliminating associated donor-site morbidity. In addition to dHACM, a number of allograft strategies are underdevelopment, including biologic grafts (porcine and cadaveric skin), synthetic material (hydrocolloids and hydrogels), and biosynthetics (Integra, Apligraf, and Dermagraft). To date, few randomized-controlled trials have compared the efficacy of these allografts in pediatric burns. Cadaveric skin has posed a number of obstacles not limited to potential disease transmission and psychologic rejection of donor skin due to potential differences in skin tone. In a longitudinal assessment of Integra in primary burn management, Integra resulted in better cosmesis compared with autograft–allograft treatment.^57^ Many of these products involve only a dermal layer; patients must still be autografted after treatment due to the lack of an epidermal layer. Apligrapf contains an epidermal layer, but its major adverse effect in clinical trials was a twofold increased wound infection rate.^58^ In addition, Apligraf has a short shelf life of only 15 days.^58^

We specifically chose dHCAM as a burn graft for patients with injuries to areas vulnerable to functional deficit from scarring such as hands, feet, face, genitalia, and joints. Although there is no limit to the body surface area that can be treated with dHACM, its cost prohibited us from using it except in these vulnerable areas. We anticipate that we could have achieved excellent results using dHCAM in all areas; however, we chose less costly dressings if we could achieve burn surface area coverage that would not compromise function due to scarring. We have shown that treatment with dHACM is associated with an accelerated rate of healing compared with STSG with little to no scarring in partial thickness wounds and less scaring in deep and full-thickness burns compared with STSG. The return to normally functioning skin including dynamic compliance, movement, and color is superior in dHACM grafts. The minimal hypertrophic scarring that did occur responded to compression therapy. Pain control was achieved without narcotics in all patients. Infectious complications were absent with the exception of one late localized fungal rash that responded to antifungal ointment. Overall, there was a reduced need for follow-up procedures or clinic visits postop compared with what might be expected from STSG. We will need to continue to follow these children longer term to describe the function and durability of the new tissue dHACM has stimulated.

The ability to regain function especially of sensitive regions such as the hands, face, feet, and genitalia is a major concern in the management of pediatric burns. While autografts ultimately lead to reconstitution of skin overlying these areas, they have also been associated with significant scarring at both primary and donor sites, which limits functional outcomes and many times requires revisional surgeries.^5^ In agreement with other studies, our results provide further support, indicating that the use of dHACM reduces scar tissue formation and enhances wound healing (Figs. 1–3).^7,38–40^ In the setting of complex wounds, chronic inflammation and aberrant immunity are catalysts for scar formation.^12,16,17^ Compared with autografts and other allografts, dHACM provides numerous epithelial growth factors and inflammatory mediators that are highly correlated with the physiologic process of changing wound healing environment from chronic inflammation to tissue regeneration.^43–47^ These changes likely account for the mitigation of scar formation after application of dHACM.

Our case series is not without limitations. First, our study was constrained by the lack of a quantitative wound depth measurement. All wounds were sorted into superficial partial, deep partial, and full thickness based on clinical assessment with the depth of burn injury being the primary indication for dHACM graft. Our bias was to treat sites with dHACM that were vulnerable to functional and cosmetic impairment due to scarring.

Second, randomized studies need to be completed to demonstrate the differences in outcomes using split thickness skin graft, other skin substitutes, and dHACM to manage complex burn wounds. Without randomization, it is difficult to demonstrate if there is more rapid healing, fewer complications, and decreased morbidity and mortality by using dHACM. However, the current barrier to widespread use of dHACM is the cost to procure dHACM and poor third-party insurance coverage. Autografts are the accepted standard of care and are available at no additional financial cost to the hospital system; yet do cost significant morbidity to the children treated. We predict that in a decision analysis, the use of dHACM, which eliminates donor-site morbidity and minimizes scar formation, may justify the upfront cost and reduce cost over time by mitigating the subsequent need for reconstructive procedures. Furthermore, the need to transfer patients with small and moderate TBSA deep partial or full-thickness wounds to dedicated burn centers represents both an additional cost and prolonged time to treatment. dHACM can be conveniently stored for rapid application and successful treatment outside of burn centers, ultimately decreasing burn center patient burden and health care costs associated with transfer of care from one system to another.

Collectively, our study suggests that dHACM provides an alternative to the current use of STSG, and is a safe, feasible, and potentially superior substitute for the management of small to moderate TBSA partial and full-thickness pediatric burns.

## Innovation

The management of pediatric burns depends on the severity of burn but typically includes surgical debridement of nonviable tissue followed by STSG.^5,6^ In the absence of adequate treatment, burns can result in significant scarring, contracture, and loss of function.^5^ We chose dHCAM as a burn graft for patients with injuries to areas vulnerable to functional deficit from scarring such as hands, feet, face, genitalia, and joints. dHACM is associated with an accelerated rate of healing and overall less scarring compared with STSG due to many tissue regenerative factors, such as growth factors and immune modulators.

**Key Findings**

- dHACM provides an alternate burn wound graft, which enhances wound healing and reduces scar tissue formation.
- dHACM augments the patient's own immunity reducing risk of infection, yet we found one patient developed an yeast infection over time due to moist wound conditions. Otherwise, there was no adverse immune reaction during the use of the dHACM.
- dHCAM has a 5-year shelf life, and is easy to apply and secure decreasing need for dressing changes during the initial 7 days of therapy.
- High patient satisfaction with once weekly dressing changes and little to no pain as scored on Baker-Wong FACES scale.
- While dHACM can be a feasible substitute for treating pediatric burns, there is on going long term follow up for these burns at this time. Randomized controlled trials of dHACM vs. other modalities have not been generated to date. This serves as an area of future research.

## About the Authors

**Natasha Ahuja, MD,** is a third-year general surgery resident at University of Buffalo. She is the first author of this article and the corresponding author. **Richard Jin, PhD,** is a fourth-year medical student at University of Buffalo who has also completed his PhD in immunology. **Colin Powers, MD**, is a fifth-year general surgery resident who completed a pediatric surgery research fellowship at University of Buffalo. **Alexandria Billi, ANP**, is one of the nurse practitioners who assists with the wound care clinic at Oishei's hospital. She has seen all the children from the beginning of the wound care until the end. **Kathryn Bass, MD, MBA,** is a pediatric surgeon with >20 years of experience. She is an associate professor of surgery at the Jacobs School of Medicine at the University of Buffalo and director of the Level 1 Pediatric Trauma Center and codirector of the Pediatric Wound Care Center at Oishei Children's Hospital. She is a fellow of the American College of Surgeons and belongs to many professional organizations, including the American Pediatric Surgical Association, The American Association for the Society of Trauma, and the International Society for Pediatric Wound Care.

## Abbreviations and Acronyms

dHACM: dehydrated human amnion chorion membrane
HTS: hypertrophic scarring
LE: lower extremity
LLE: left lower extremity
LUE: left upper extremity
OR: operating room
PIP: proximal interphalangeal
RUE: right upper extremity
STSG: split thickness skin graft
TBSA: total body surface area
VSS: Vancouver Scar Scale
